# Outcomes of a randomized controlled trial assessing a smartphone *A*pplication to reduce unmet needs among people diagnosed with *C*anc*E*r (ACE)

**DOI:** 10.1002/cam4.2718

**Published:** 2019-11-25

**Authors:** Patricia M. Livingston, Leila Heckel, Liliana Orellana, David Ashley, Anna Ugalde, Mari Botti, Graham Pitson, Anne Woollett, Suzanne K. Chambers, Phillip Parente, Jacqueline Chirgwin, Cathrine Mihalopoulos, Barbara Lavelle, Jennifer Sutton, Jo Phipps‐Nelson, Mei Krishnasamy, Katherine Simons, Natalie Heynsbergh, Nilmini Wickramasinghe, Vicki White

**Affiliations:** ^1^ Faculty of Health Deakin University Geelong Vic. Australia; ^2^ School of Nursing and Midwifery Faculty of Health Deakin University Geelong Vic. Australia; ^3^ Biostatistics Unit Faculty of Health Deakin University Geelong Vic. Australia; ^4^ Duke University School of Medicine Durham NC USA; ^5^ Epworth HealthCare Richmond Vic. Australia; ^6^ Andrew Love Cancer Centre Barwon Health University Hospital Geelong Vic. Australia; ^7^ The University of Melbourne, Medicine, Dentistry and Health Sciences Melbourne Vic. Australia; ^8^ Faculty of Health University of Technology Sydney Sydney NSW Australia; ^9^ Faculty of Medicine, Nursing and Health Sciences Monash University Clayton Vic. Australia; ^10^ Eastern Health Department of Oncology Box Hill Vic. Australia; ^11^ Faculty of Health School of Health and Social Development Deakin University Geelong Vic. Australia; ^12^ Consumer Melbourne Vic. Australia; ^13^ Peter MacCallum Cancer Centre Melbourne Vic. Australia; ^14^ North Eastern Melbourne Integrated Cancer Service Heidelberg Vic. Australia; ^15^ Iverson Health Innovation Research Institute Swinburne University Hawthorn Vic. Australia; ^16^ Faculty of Health School of Psychology Deakin University Geelong Vic. Australia

**Keywords:** cancer education, clinical cancer research, smartphone technology, translational research

## Abstract

**Background:**

Smartphone technology represents an opportunity to deliver practical solutions for people affected by cancer at a scale that was previously unimaginable, such as information, appointment monitoring, and improved access to cancer support services. This study aimed to determine whether a smartphone application (app) reduced the unmet needs among people newly diagnosed with cancer.

**Methods:**

A single blind, multisite randomized controlled trial to determine the impact of an app‐based, 4‐month intervention. Newly diagnosed cancer patients were approached at three health service treatment clinics.

**Results:**

Eighty‐two people were randomized (intervention; n = 43 and control; n = 39), average age was 59.5 years (SD: 12.9); 71% female; 67% married or in a de facto relationship. At baseline, there were no differences in participants’ characteristics between the groups. No significant effects, in reducing unmet needs, were demonstrated at the end of intervention (4‐month) or 12‐month follow‐up. Overall, 94% used the app in weeks 1‐4, which decreased to 41% in weeks 13‐16. Mean app use time per participant: Cancer Information, 6.9 (SD: 18.9) minutes; Appointment Schedule, 5.1 (SD: 9.6) minutes; Cancer Services 1.5 minutes (SD: 6.8); Hospital Navigation, 1.4 (SD: 2.8) minutes.

**Conclusions:**

Despite consumer involvement in the design of this smartphone technology, the app did not reduce unmet needs. This may have been due to the study being underpowered. To contribute to a meaningful understanding and improved implementation of smartphone technology to support people affected by cancer, practical considerations, such as recruitment issues and access to, and confidence with, apps, need to be considered.

Australian New Zealand Clinical Trials Registration (ACTRN) Trial Registration: 12616001251415; WEF 7/9/2016.

## Graphical abstract

Smartphone technology represents an opportunity to deliver practical solutions for people affected by cancer at a scale that was previously unimaginable, such as information, and improved access to cancer support services.

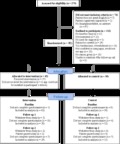

## 1 INTRODUCTION

With improved diagnostic and treatment options, seven in 10 people are surviving cancer beyond 5 years and over one million people in Australia are currently survivors of cancer.^1^ People diagnosed with cancer experience moderate levels of both distress and unmet needs during treatment and into the survivorship phase of their condition. It is well‐established that information provision and support is essential to high quality care and results in psychosocial benefits to people diagnosed with cancer.^2^ While distress can be prevalent throughout the cancer trajectory, high levels are found particularly at diagnosis and during active cancer treatment.^3^, ^4^, ^5^ Mehnert and colleagues, using the distress thermometer (DT), reported high levels of distress symptoms (DT >4) in 52% of cancer patients with the prevalence increasing to 81% in the presence of sleep problems, fatigue, and difficulties getting around.^4^ Previous studies have also demonstrated that high levels of emotional distress are associated with decreased quality of life, high symptom burden, and poor adherence to treatment.^6^, ^7^

Despite consistent evidence and widespread acknowledgment of the psychological impact of a cancer diagnosis, unmet needs which may contribute to an individual's distress are poorly recognized and undertreated.^8^ Unmet needs refer to the gap between a person's experience of services and the actual services required or desired.^9^ A systematic review found that up to 93% of newly diagnosed patients reported unmet needs across a range of domains comprising psychological, informational, and physical.^10^ An earlier review^11^ identified that the most common unmet needs were those associated with activities of daily living, economic needs, physical needs, supportive care needs, and sexuality. This situation has not changed in nearly a decade with similar patterns and predictors of unmet needs reported by Okediji and colleagues in 2017.^12^

With the rapid development of information science and technology, digital health has become an important tool for health care. As a result, many people seek health‐related information on the Internet.^13^, ^14^ However, concerns over the quality of information^14^ and level of e‐health literacy^15^ highlight the need to provide people affected by cancer with quality and flexible access to information from reputable sources.

Smartphone technology represents an opportunity to deliver practical solutions at a scale that was previously unimaginable. It offers new possibilities for health promotion and treatment management that can reach patients regardless of where they live, including appointment monitoring,^16^, ^17^ improved access to cancer information and support services,^18^ and lead to improve their participation in health care over time.^19^, ^20^

To inform the development of the smartphone application (app), we undertook focus groups to determine the type of features that would be important to include in an app. Based on consumer feedback, the smartphone app prototype, referred to as the ACE app, was developed with partners, Barwon Health, Simble Solutions^©^ and Optus^©^. ACE was a free to user, downloadable application to a smartphone or similar tablet device designed for people affected by cancer. It provided access to a list of appointments from their treating health service; targeted information on patient's cancer type; increased awareness and access to the Cancer Council Victoria's Cancer Information and Support Service (CISS) that linked people to a range of community‐based supportive care services as required; and identification of distress. Those with elevated levels of distress were signaled by IT services and referred to CISS with a name and contact telephone number for referral to psychological services.

The ACE app was piloted with a convenience sample of 20 people newly diagnosed with cancer. The app recorded 250 hits in the first month alone, suggesting the app would be acceptable to, and useful for, this population group.

The current study aimed to assess through a randomized controlled trial whether access to the ACE app for a 4‐month period reduced unmet needs during the treatment period. Secondary aims were to determine whether the app reduced distress and was used by participants. We tested the hypotheses that access to the ACE app would reduce unmet needs (with particular reference to informational needs during treatment) and levels of distress.

## 2 MATERIALS AND METHODS

### 2.1 Research design

A single blinded, multisite randomized controlled trial to determine the efficacy of the ACE smartphone app intervention delivered for 4 months among newly diagnosed cancer patients. Assessments occurred at baseline, and 4, and 12 months following recruitment.

#### 2.1.1 Setting

The study was undertaken across one private and two public health services or hospitals in Victoria Australia. Participating health services were situated in urban and rural areas with socially diverse patient populations and similar IT operating systems. The health‐care system in Australia comprises both government (public health services) and private hospitals that are owned and managed privately but licensed and regulated by the Government. Recruitment occurred between February 2017 and January 2018.

#### 2.1.2 Procedures

Newly diagnosed cancer patients were identified and screened for eligibility by medical or nurse clinicians at each of the three health services. Research trained personnel were provided with a list of eligible patients who were then approached in the outpatient oncology setting. Each patient was given a brief introduction to the study. Interested patients received a study pack (consent form and questionnaire) to take home and were then followed‐up over the phone within 48 hours by the research project manager to answer any questions and to confirm participation. Patients then completed and returned the written informed consent and baseline questionnaire using a reply‐paid envelope. Following return of the consent form, participants were allocated to the study arm based on computer‐generated randomization sequences, stratified by health service and patient's age, produced by the trial statistician.

For those allocated to the intervention, research personnel at each health service downloaded the app onto the participant's smartphone or similar tablet device and showed features of the app at their next treatment appointment. Participants allocated to the control group received usual care, from their treating health service.

#### 2.1.3 Participant inclusion criteria

Adults, aged 18+ years, with a new cancer diagnosis; with curative intent (stages 1‐3) and attending outpatient treatment settings for cycle 2‐5 of adjuvant chemotherapy or fraction 2‐5 for radiotherapy treatment; able to complete English language questionnaires; and having access to a smartphone or similar tablet device. Exclusion criterion was cognitive dysfunction (psychotic illness or dementia) as determined by experienced health service oncology nurses.

### 2.2 Intervention

The smartphone app comprised the following features:

1. Login/ logout;
2. Cancer information, links to reputable cancer sites, for example, the National Cancer Institute, Cancer Institute NSW (eviQ), Cancer Council Victoria (CCV) Treatment, Lifestyle and Emotion, and Cancer booklets;
3. Information on the CCV and support service;
4. Health Service Navigation, provision of maps to enable participants to navigate their way around their health service, and information on transport, parking, facilities and services available;
5. Information on allied services at each health service, for example nutrition and dietetics, speech pathology, psychology, social work, physiotherapy;
6. Appointment schedule. The ACE app was linked into the health service IT system, showing a list of upcoming treatment appointments and facilitated requests to re‐schedule existing bookings (triggered an automatic email to the clinic receptionist at the health service);
7. Distress Thermometer^21^ with a prompt to complete it at baseline and monthly thereafter for 4 months. The DT is a one‐item screening tool for the detection of generalized distress, with scores from 0 (no distress) to 10 (extreme distress). Scores 4 to 6 prompted a notification to the participant, recommending they contact CISS and phone number; scores of ≥7 triggered an automatic request to the participant whether they would like a cancer nurse to contact them from CISS. If the participant responded yes, an email (containing the participant's contact information and DT score) was sent to CISS for follow‐up with the participant within 4 hours. If the participant declined the referral, emergency telephone numbers were provided.
8. Links to clinical trials information fact sheets from CCV and contact details of clinical trial coordinators at each health service;
9. A Notebook for writing down questions prior to consultations; and
10. Emergency telephone numbers if needed during out of business hours.

While the app comprised 10 features, only the Appointment schedule, DT, and Notebook required login so the navigational format was flexible depending on the participant's needs to support sustained use over the intervention period.^22^

### 2.3 Data collection

Questionnaires were distributed by mail with a reply‐paid envelope to return to the project manager. Demographic and clinical characteristics were collected at baseline and included information on participant age, gender, postcode, education, cultural background, employment status, living situation, marital status, cancer type, stage and current treatment.

### 2.4 Outcome measures

#### 2.4.1 Primary outcome

Unmet needs were ascertained using the *Supportive Care Needs Survey (*SCNS‐SF34) which measures the unmet needs of cancer patients across the illness trajectory. This questionnaire comprises the following five domains: health system and information needs; physical and daily living needs; psychological needs; sexuality needs; and patient care and support needs. The scale has been validated with cancer patients, is reliable, and has demonstrated good content and construct validity for measuring global needs in cancer patients.^23^, ^24^

#### 2.4.2 Secondary outcomes

*Impact of Events Scale* (IES‐R). The IES‐R is an index of cancer‐specific distress. This 22‐item scale evaluates stress reactions after traumatic experiences such as having been diagnosed with cancer. Responses are made on a 5‐point Likert scale ranging from 0 (“not at all”) to 4 (“extremely”). Higher score indicates greater stress. The IES‐R comprises three subscales, namely intrusion, avoidance, and hyper‐arousal. The IES‐R has been used frequently to assess the distress following a cancer diagnosis and has been found to have good internal consistency.^25^

*Health Education Impact Questionnaire* was designed to evaluate the intended benefits of a wide variety of self‐management programs. It is used in over 20 countries and has been adapted to the cancer setting.^26^ It contains 40 questions across eight scales, each with high reliability. Two highly reliable scales, Health Service Navigation, which measures the confidence and ability to communicate and negotiate with health service providers, and Emotional Distress, which measures negative affective responses reflected the intended outcomes of the intervention.

*Health Literacy Questionnaire* measures a person's capacity to seek, understand, and use health information. The tool provides insight into client‐practitioner interactions, guides program redevelopment and organizational responses to populations with low health literacy, specifically “having sufficient information to manage my health”; “actively managing my health”; “ability to actively engage with health‐care providers”; “navigating the health‐care system”; and the “ability to find good health information.”^27^

*Satisfaction with the ACE app*. Information on the usefulness and acceptability of the app was obtained from participants in the intervention arm at the first follow‐up. The questionnaire included questions about participants’ expectations of the app and what features of the app were or were not taken up.

*App usage* was extracted from data downloaded from a cloud‐based server, hosted by the developers of the app.

### 2.5 Sample size calculations

The main outcome was a reduction in unmet needs as measured by each of the five domains of the SCNS‐SF34. We planned to use linear mixed models with three measurement points (baseline, 4, and 12 months), and the *F*‐test to assess the study arm by time interaction (*α* = .05). Assuming a standard deviation of 30 and an intraclass correlation coefficient of 0.2 (or as high as 0.8), a study with 125 patients evaluated three times in each arm had 80% power to detect a reduction of 14.5 (or 7.25) at 4 months and maintained (if not increased) at 12 months. Reductions in this range were comparable to the significant differences (7.7‐15.0), reported by Boyes et al,^24^ between cancer patients in, and not in, remission, for 5 domains of the SCNS‐SF34.

### 2.6 Statistical analysis

Baseline characteristics of participants allocated to the two conditions were compared using the Chi‐square or Fisher's exact test for categorical variables and *t* test for numerical variables. All analyses were based on an intention‐to‐treat (ITT) approach. The effect of the intervention on each of the outcomes was estimated using generalized estimating equations (GEE) with an exchangeable working correlation matrix to account for the repeated measures. Models included group (intervention/control), time (baseline, 4, and 12 months), and the interaction study arm × time. No adjustment to the significance level for multiplicity of endpoints was performed. Effect of the intervention on number of moderate to high needs (score of 4 to 5) was also assessed. All analyses were performed with SAS software, version 9.4 (SAS Institute).

## 3 RESULTS

**Figure 1:**
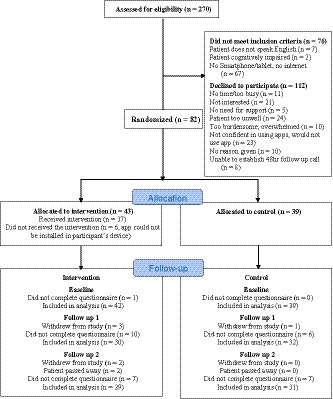
Consort diagram showing recruitment of participants into the study

A total of 270 people were screened for eligibility, of which 76 (28%) were deemed ineligible. Overall, 112 (41%) people declined to participate, the reasons are provided in Figure 1. A total of 82 people agreed to take part, the average age was 59.5 years (SD: 12.9); 71% were female; the majority were married or living in a de facto relationship (67%); 34% had a tertiary qualification; over half of the sample had breast cancer. Participants were randomized into intervention (n = 43) and control (n = 39) groups. Participants’ demographic characteristics are provided in Table 1. At baseline, there were no significant differences in participants’ demographic or clinical characteristics between the two groups. The overall attrition rate was 24% (intervention, 30%; control, 18%).

**Table 1:** Demographic and clinical characteristics of participants in the ACE trial at baseline by condition (N = 82)

Characteristics	All	Control (n = 39)	Intervention (n = 43)	*P*‐value
Age, mean (SD), y	59.5 (12.9)	58.8(13.7)	60.1 (12.4)	.64^a^
Age groups, n (%), (y)				
18‐49	20 (24)	10 (26)	10 (23)	.97^b^
50‐59	15 (18)	7 (18)	8 (19)	
60	47 (57)	22 (56)	25 (58)	
Gender, n (%)				
Male	24 (29)	13 (33)	11 (26)	.44^b^
Female	58 (71)	26 (67)	32 (74)	
Marital status, n (%)				
Single	8 (10)	4 (10)	4 (9)	.22^c^
Married	49 (60)	21 (54)	28 (65)	
De facto	6 (7)	3 (8)	3 (7)	
Separated	2 (2)	1 (3)	1 (2)	
Divorced	9 (11)	3 (8)	6 (14)	
Widowed	8 (10)	7 (18)	1 (2)	
Education, n (%)				
Secondary school	22 (27)	11 (28)	11 (26)	.54^c^
Certificate or diploma	25 (31)	10 (26)	15 (35)	
University degree	28 (34)	13 (33)	15 (35)	
Other	7 (9)	5 (13)	2 (5)	
Employment, n (%)				
Working full time	20 (26)	10 (26)	10 (25)	.59
Working part time	15 (19)	8 (21)	7 (17)	
Retired	30 (39)	12 (32)	18 (45)	
Other	12 (16)	8 (21)	5 (13)	
Living situation, n (%)				
Living by myself	17 (21)	10 (26)	7 (16)	.39^c^
Living with partner/spouse and/or family	60 (73)	27 (69)	33 (77)	
Living with friends	4 (5)	1 (3)	3 (7)	
Other	1 (1)	1 (3)		
Type of cancer, n (%)				
Breast	50 (61)	21 (54)	29 (67)	.69^c^
Prostate	2 (2)	1 (3)	1 (2)	
Colorectal	8 (10)	4 (10)	4 (9)	
Lung	4 (5)	3 (8)	1 (2)	
Others	22 (27)	13 (33)	9 (21)	

a *t* test.

b Chi‐squared test.

c Fisher's exact test.

Table S2 presents the estimated mean for SCNS‐SF34, unmet needs and IES‐R outcomes for each measurement time and the differences in change from baseline between intervention and control groups. There were no significant differences between the groups in any of the outcomes. A downward trend for “physical and daily living needs” and “psychological needs” was observed from the SCNS‐SF34 over time and a downward trend to lower total distress scores in the intervention group at the 4 and 12 months follow‐up was observed from the IES‐R. Table S2 displays the same estimates for the other secondary outcomes.

### 3.1 ACE app usage

Of the 43 participants randomized to the intervention group, the app could not be downloaded onto their devices for six participants due to technical issues. Findings from data analytics showed that over the 4‐month trial period, 34 (of 43 randomized to the ACE‐app arm) participants accessed the ACE app (Table S3); 32 (94%) used the app in the first 4 weeks of the intervention which decreased to 20 (59%) in weeks 5‐8 and 9‐12, with 14 (41%) in the final 13‐16 weeks. Twenty (59%) participants logged in more than five times over the 16‐week period, and 17% logged in three to four times. The most common features accessed were: Cancer Information (230 minutes; mean use per participant: 6.9 (SD 18.9) minutes), Appointments (173.9 minutes; mean use per participant: 5.1 (SD 9.6) minutes), CISS services (49.9 minutes; mean use per participant 1.5 (SD: 6.8) minutes), and Hospital navigation (48.3 minutes; mean use per participant 1.4 (SD: 2.8) minutes).

A total of 79 DT scores were recorded. Medium to high distress levels were recorded by 44% participants at baseline, 44% of participants at the second monthly assessment, 46% at the third monthly assessment, and 40% at the final assessment.

### 3.2 ACE app feedback

Nearly two‐thirds of participants (63%) reported that they strongly agreed or agreed that people would learn to use the app very quickly; 74% of participants reported they found the DT easy to complete on the app; 61% reported feeling confident or very confident using the app, with only one person reporting not being confident using the app.

Representative feedback from participants included comments that the app was “a good idea though as I found it confronting how much paper material/info I was bombarded with”; “was easy to navigate to information and applicable material of interest (when connectivity issues actually enabled access)”; “I found appointments and some available services useful”; “while suffering from side effects of chemo then [the] app didn't help. At pain‐free times information was useful”; “The developers are on a great path—keep going and well done and thank you.”

## 4 DISCUSSION

There has been increasing interest in mobile phones as a platform for interventions for people with cancer.^28^ A recent systematic review highlighted mobile interventions for people with cancer only met treatment or symptom‐related information needs and did not meet patients' full range of cancer‐related information needs, from information on psychological support to how to manage finances during cancer, and the long‐term effects of treatment.^28^ Our smartphone app intervention attempted to fill this gap by providing information, support services, clinical trials information, and allied health resources to people newly diagnosed with cancer.

This smartphone app was designed to facilitate opportunities for improving person‐centered care using mobile technology. While the ACE app was well‐received and utilized by participants, our study did not find it effective in reducing unmet needs among newly diagnosed cancer patients. It may be that current measures of unmet need are not sufficiently sensitive to consistently identify small changes that reflect the particular needs of an individual.^29^ However despite the lack of significant difference, the pattern of results for distress was encouraging with lower overall IES score for the intervention group compared to the control condition at both follow‐up measures. The smaller than expected number of recruited participants, 82 instead of the projected 250 participants are likely to have limited the power of the study. The low recruitment was due to several issues relating to hospital IT systems which influenced the start date and participation of health services. At one health service, negotiations regarding IT firewalls introduced substantial delays in the project start date by 9 months, while relocation of another large cancer‐focused health service resulted in ongoing IT issues at this site; it became apparent that recruitment of the planned number of patients for that particular site (n = 80) could not be achieved within the project time line.

Participants reported that the ACE app was acceptable in terms of ease of learning and DT completion. On average, participants recorded 2.3 of the recommended 4 DT scores, and for those who scored high distress levels and agreed to be referred were contacted by CISS for follow‐up support. This app has the potential to reduce the burden of screening and follow‐up in busy clinics for those flagged who were highly distressed, leaving clinicians free to focus on those who need specialized follow‐up.

The results also showed that while usage was highest during the first month of the intervention, usage decreased thereafter. The most accessed features were Cancer Information, Appointments, CCV services, and Health service navigation. A recent survey of hematology‐oncology patients also reported interest in having access to health‐related information via mobile apps with appointment management, advice on disease management, and communication with health professionals.^30^ The ACE app contained several of these features; however, it did not have an interactive component with health‐care providers which may have had a more positive impact on patient outcomes, had symptom management or concerns as part of managing their disease been available.^30^ Moreover, engagement with self‐guided interventions can be low.^31^

### 4.1 Study limitations

The current study was a randomized controlled study which provided precise measures of efficacy under ideal research conditions, resulting in results being less prone to bias.^32^ The design also involved consumers from the early design stages with regular input and feedback. While collecting data on usage was a strength of our study, a limitation was that we could only capture data usage when the participant was logged into the app. As features such as information, CISS services, health service navigation, and clinical trials information could be accessed without logging onto the app, our count of access to these services may be an under‐estimate. We did not anticipate Internet variability across hospital sites, with black spots located across different clinics, which resulted in downloading issues for some participants. Moreover, financial funding constraints did not allow for an extension of the recruitment period post the original recruitment timeline of 12 months.

Our recruitment rate of 42% was consistent with previous research where recruitment among this population can vary from 20% to 60% for technology‐based intervention studies.^33^ Sixty‐seven (25%) patients approached did not have a smart phone or similar tool, which was low compared to estimates of over 50% of people over the age of 65 years using smartphones or tablets.^34^ Despite our study including a staff member demonstrating how to use the ACE app, a fifth of eligible patients declined participation because they did not feel confident in using apps or did not think they would use an app. This suggests that while smart phones might be widespread, their functionality may not be fully utilized by users including older users. In addition, nearly two‐thirds of our sample had a breast cancer diagnosis. As this patient population may be better serviced compared to other cancer groups, further work is required to test the usefulness of health apps in studies that ensure inclusion of a broad representation of people affected by cancer.^35^

### 4.2 Clinical implications

The practical difficulties in undertaking smartphone technology research are highlighted by this study. The practicality of apps may not match the reality in that people may use such apps initially but tend not to continue to use them consistently for longer periods. Providing additional information such as survivorship features or sharing the app with carers may facilitate ongoing use. Undertaking technology‐based research in hospital settings can be problematic with Internet black spots, firewalls and technology incompatibility, posing substantial impediments to the conduct of studies.^36^

### 4.3 Conclusion

Expanding evaluation methodologies, that ensure robust study designs while allowing practical issues to be considered will contribute to a meaningful understanding and improved implementation of smartphone applications.^37^

## CONFLICT OF INTEREST

The authors declare that they have no competing interests.

## AUTHORS' CONTRIBUTIONS

PML, LH, LO, AU, MB, SKC, JC, CM, JS, KS, PP, and VW conceptualized and designed the study; PML, LH, LO, and BL collected and assembled the data; PML, LH, LO, NH, AU, MB, SKC, JC, CM, JS, KS, PP, and VW performed data analysis and interpretation; all the authors involved in manuscript writing.
