# Supplementary material for: Outcomes of a randomized controlled trial assessing a smartphone Application to reduce unmet needs among people diagnosed with CancEr (ACE)
Source: Cancer Med. 2019 Nov 25;9(2):507–16. doi: 10.1002/cam4.2718 (PMC6970035; doi:10.1002/cam4.2718)
Supplement: Supplementary file 1 [file CAM4-9-507-s001.docx]

Table 2. Outcomes at baseline, end of intervention (4 months) and follow up (12 months) in the two groups.

|  | **Measurement**  **Time** | **Control**  **Mean (95% CI)** | **Intervention**  **Mean (95% CI)** | **Time × Group Interaction P** | **Change from baseline Intervention vs Control**  **Mean difference (95% CI)** |
| --- | --- | --- | --- | --- | --- |
|  |  |  |  |  |  |
| *Supporting Care Needs Survey* | Baseline | 22.4 (17.6, 27.2) | 29.0 (23.5, 34.5) | 0.3911 |  |
| Health System and information needs | 4 months | 19.1 (13.7, 24.5) | 19.9 (14.2, 25.7) |  | -5.79 (-14.16, 2.59) |
|  | 12 months | 13.5 (8.5, 18.6) | 15.9 (10.8, 21.0) |  | -4.21 (-12.96, 4.53) |
|  |  |  |  |  |  |
| *Supportive Care Needs Survey* | Baseline | 35.5 (27.8, 43.3) | 34.2 (28.1, 40.3) | 0.9304 |  |
| Physical and daily living needs | 4 months | 29.3 (20.5, 38.2) | 28.8 (20.4, 37.2) |  | 0.76 (-12.01, 13.54) |
|  | 12 months | 24.8 (16.9, 32.8) | 21.9 (14.5, 29.3) |  | -1.64 (-12.24, 8.96) |
|  |  |  |  |  |  |
| *Supportive Care Needs Survey* | Baseline | 29.0 (21.5, 36.6) | 31.7 (25.6, 37.8) | 0.3112 |  |
| Psychological needs (10 items) | 4 months | 32.1 (22.8, 41.5) | 30.5 (23.3, 37.8) |  | -4.25 (-15.71, 7.20) |
|  | 12 months | 21.7 (14.3, 29.1) | 17.8 (11.6, 23.9) |  | -6.63 (-14.95, 1.68) |
|  |  |  |  |  |  |
| *Supportive Care Needs Survey* | Baseline | 14.5 (7.6, 21.4) | 20.7 (12.5, 28.8) | 0.5017 |  |
| Sexuality needs | 4 months | 16.2 (8.4, 24.1) | 16.0 (8.6, 23.4) |  | -6.35 (-16.80, 4.11) |
|  | 12 months | 14.6 (6.9, 22.2) | 17.4 (9.0, 25.7) |  | -3.31 (-13.66, 7.03) |
|  |  |  |  |  |  |
| *Supportive Care Needs Survey* | Baseline | 17.2 (13.9, 20.4) | 19.6 (15.2, 24) | 0.7807 |  |
| Patient care and support needs | 4 months | 13.0 (9.4, 16.5) | 18.1 (11.6, 24.6) |  | 2.7 (-4.98, 10.38) |
|  | 12 months | 8.6 (4.8, 12.3) | 11.5 (6.9, 16.2) |  | 0.57 (-6.19, 7.33) |
|  |  |  |  |  |  |
| *Number of moderate to high unmet* | Baseline | 4.03 (2.31, 5.74) | 4.14 (2.53, 5.76) | 0.5523 |  |
| *needs (yes=4 to 5)* | 4 months | 4.47 (2.82, 6.13) | 3.26 (1.57, 4.95) |  | -1.33 (-3.67, 1.01) |
|  | 12 months | 2.25 (0.96, 3.54) | 1.87 (0.50, 3.25) |  | -0.49 (-2.76, 1.78) |
|  |  |  |  |  |  |
| *Impact of Event scale (IESR)* | Baseline | 20.5 (16.4, 24.5) | 19.2 (15.2, 23.2) | 0.8314 |  |
| Total Score | 4 months | 20.4 (16.0, 24.9) | 17.7 (14.0, 21.5) |  | -1.40 (-6.13, 3.32) |
|  | 12 months | 17.6 (12.7, 22.6) | 15.3 (11.2, 19.5) |  | -0.99 (-5.67, 3.69) |
|  |  |  |  |  |  |
| *Impact of Event scale (IESR)* | Baseline | 7.2 (5.4, 9.0) | 6.8 (5.2, 8.4) | 0.9473 |  |
| Intrusion subscale | 4 months | 7.5 (5.6, 9.3) | 6.8 (5.1, 8.5) |  | -0.33 (-2.45, 1.78) |
|  | 12 months | 6.7 (4.7, 8.6) | 6.1 (4.3, 7.8) |  | -0.25 (-2.37, 1.86) |
|  |  |  |  |  |  |
| *Impact of Event scale (IESR)* | Baseline | 8.6 (7.1, 10.2) | 8.2 (6.4, 10.1) | 0.9933 |  |
| Avoidance subscale | 4 months | 8.0 (6.1, 9.9) | 7.6 (5.7, 9.5) |  | 0.01 (-2.24, 2.26) |
|  | 12 months | 6.6 (4.6, 8.7) | 6.4 (4.2, 8.5) |  | 0.13 (-2.09, 2.34) |
|  |  |  |  |  |  |
| *Impact of Event scale (IESR)* | Baseline | 4.6 (3.2, 6.1) | 4.1 (3, 5.3) | 0.6642 |  |
| Hyperarousal subscale | 4 months | 4.7 (3.2, 6.2) | 3.4 (2.3, 4.5) |  | -0.79 (-2.60, 1.02) |
|  | 12 months | 3.7 (2.4, 5.1) | 2.9 (1.8, 4.1) |  | -0.25 (-2.02, 1.52) |
|  |  |  |  |  |  |
|  |  |  |  |  |  |
| *Health Literacy Questionnaire* | Baseline | 3.18 (2.96, 3.4) | 3.33 (3.17, 3.48) | 0.1098 |  |
| S1 Healthcare provider support | 4 months | 3.37 (3.18, 3.56) | 3.19 (3.03, 3.34) |  | -0.33 (-0.63, -0.03) |
|  | 12 months | 3.43 (3.23, 3.62) | 3.35 (3.09, 3.62) |  | -0.22 (-0.61, 0.16) |
|  |  |  |  |  |  |
| *Health Literacy Questionnaire* | Baseline | 2.65 (2.46, 2.85) | 2.59 (2.42, 2.75) | 0.6925 |  |
| S5 Critical appraisal | 4 months | 2.62 (2.43, 2.81) | 2.65 (2.51, 2.79) |  | 0.1 (-0.23, 0.42) |
|  | 12 months | 2.68 (2.48, 2.89) | 2.6 (2.36, 2.85) |  | -0.01 (-0.37, 0.34) |
|  |  |  |  |  |  |
| *Health Literacy Questionnaire* | Baseline | 4.23 (4.07, 4.38) | 4.28 (4.14, 4.42) | 0.4358 |  |
| S6 Active engagement with | 4 months | 4.25 (4.06, 4.43) | 4.14 (3.99, 4.3) |  | -0.16 (-0.43, 0.11) |
| healthcare providers | 12 months | 4.42 (4.24, 4.59) | 4.33 (4.16, 4.5) |  | -0.14 (-0.4, 0.11) |
|  |  |  |  |  |  |
| *Health Literacy Questionnaire* | Baseline | 4.03 (3.87, 4.18) | 4.1 (3.95, 4.25) | 0.3892 |  |
| S7 Navigating the healthcare system | 4 months | 4.09 (3.9, 4.27) | 3.99 (3.79, 4.18) |  | -0.17 (-0.42, 0.08) |
|  | 12 months | 4.18 (3.94, 4.41) | 4.22 (4.06, 4.38) |  | -0.04 (-0.29, 0.22) |
|  |  |  |  |  |  |
| *Health Literacy Questionnaire* | Baseline | 3.97 (3.79, 4.15) | 4.16 (3.99, 4.33) | 0.2199 |  |
| S8 Ability to find health information | 4 months | 4.05 (3.82, 4.28) | 4.05 (3.93, 4.17) |  | -0.19 (-0.48, 0.1) |
|  | 12 months | 4.26 (4.08, 4.44) | 4.19 (4.02, 4.36) |  | -0.26 (-0.56, 0.04) |
|  |  |  |  |  |  |
| *Health Literacy Questionnaire* | Baseline | 4.21 (4.06, 4.35) | 4.3 (4.16, 4.44) | 0.2209 |  |
| S9 Reading and understanding | 4 months | 4.23 (4.05, 4.42) | 4.23 (4.12, 4.35) |  | -0.1 (-0.35, 0.15) |
| health information | 12 months | 4.41 (4.25, 4.58) | 4.29 (4.14, 4.43) |  | -0.22 (-0.47, 0.03) |
|  |  |  |  |  |  |
| *Health Education Impact Questionnaire* | Baseline | 2.78 (2.60, 2.95) | 2.99 (2.75, 3.23) | 0.1472 |  |
| Health directed behaviour | 4 months | 2.89 (2.64, 3.14) | 3.03 (2.82, 3.24) |  | -0.07 (-0.38, 0.23) |
|  | 12 months | 3.24 (3.04, 3.44) | 3.17 (2.93, 3.41) |  | -0.28 (-0.57, 0.01) |
|  |  |  |  |  |  |
| *Health Education Impact Questionnaire* | Baseline | 3.08 (2.93, 3.23) | 3.18 (2.99, 3.38) | 0.6059 |  |
| Positive and active engagement in life | 4 months | 3.22 (3.01, 3.42) | 3.22 (3.05, 3.40) |  | -0.09 (-0.37, 0.18) |
|  | 12 months | 3.36 (3.21, 3.51) | 3.34 (3.16, 3.52) |  | -0.12 (-0.36, 0.12) |
|  |  |  |  |  |  |
| *Health Education Impact Questionnaire* | Baseline | 3.09 (2.95, 3.23) | 3.06 (2.88, 3.24) | 0.3991 |  |
| Skill and technique acquisition | 4 months | 3.07 (2.89, 3.25) | 3.08 (2.9, 3.27) |  | 0.04 (-0.23, 0.31) |
|  | 12 months | 3.22 (3.03, 3.41) | 3.05 (2.9, 3.21) |  | -0.14 (-0.41, 0.13) |
|  |  |  |  |  |  |
|  |  |  |  |  |  |

*All estimates, 95% confidence intervals (CI) and p-values obtained under generalized estimating equation models*
