# Supplementary material for: Outcomes of a randomized controlled trial assessing a smartphone Application to reduce unmet needs among people diagnosed with CancEr (ACE)
Source: Cancer Med. 2019 Nov 25;9(2):507–16. doi: 10.1002/cam4.2718 (PMC6970035; doi:10.1002/cam4.2718)
Supplement: Supplementary file 2 [file CAM4-9-507-s002.docx]

| Table 3. Duration and frequency of ACE App usage and reason for accessing the app (n=34) | |
| --- | --- |
| **Use of app**  Could not log in due to technical issues 6 (14%)  Did not log in at all 3 (7%)  Logged in at least once 34 (79%)  **Mean duration of use of each ACE App section per participant (n=34), reported in minutes when logged into the app (SD) over intervention period mean (SD)** | |
| Cancer information | 6.9 (18.9) |
| Appointments | 5.1 (9.6) |
| CCV services | 1.5 (6.8) |
| Hospital navigation | 1.4 (2.8) |
| Notebook | 1.3 (4.8) |
| Allied health | 0.4 (1) |
| Clinical Trials | 0.3 (0.6) |
| Help | 0.1 (0.4) |
| **Total duration of use per ACE App section over 16 weeks (minutes) when logged in to the app (n=34).** | |
| Cancer information | 230.2 |
| Appointments | 173.9 |
| CCV services | 49.9 |
| Hospital navigation | 48.3 |
| Notebook | 44.8 |
| Allied health | 14.8 |
| Clinical Trials | 10.5 |
| Help | 2.5 |
| **^*^Total frequency of logins over the intervention period (n=34)** | **n (%)** |
| 1-2 logins in 16 weeks | 8 (24) |
| 3-4 logins in 16 weeks | 6 (17) |
| 5+ logins in 16 weeks | 20 (59) |
| **^*^Frequency of logins during each month of the intervention period (total=241)** |  |
| Weeks 1-4 | 117 (49%) |
| Weeks 5-8 | 58 (24%) |
| Weeks 9-12 | 39 (16%) |
| Weeks 13-16 | 27 (10%) |
| Table 3 cont’d  **Frequency of ACE app users throughout the intervention period (n=34)** |  |
| Weeks 1-4 | 32 (94%) |
| Weeks 5-8 | 20 (59%) |
| Weeks 9-12 | 20 (59%) |
| Weeks 13-16 | 14 (41%) |
| **Total number of visits per ACE App section** | |
| Appointments | 159 |
| Cancer information | 57 |
| Notebook | 35 |
| Hospital Navigation | 33 |
| Cancer Council Victoria Support Services | 28 |
| Clinical Trials | 23 |
| Allied Health | 13 |
| Help | 3 |
| **Reason for accessing the app** | |
| Use ACE App resources only | 103 (43) |
| Use ACE App resources and complete Distress Thermometer | 79 (33) |
| Complete Distress Thermometer only | 59 (24) |

** Login was only required to access the Appointment, Distress Thermometer, and Notebook feature.*
